# (4*S*,5*S*)-2-(2-Fluoro­phen­yl)-1,3-dioxolane-4,5-dicarboxamide

**DOI:** 10.1107/S1600536808039470

**Published:** 2008-11-29

**Authors:** Xin-Hua Li, De-Cai Wang, Bo-Nian Liu, Wei Xu

**Affiliations:** a Sate Key Laboratory of Materials-Oriented Chemical Engineering, College of Life Science and Pharmaceutical Engineering, Nanjing University of Technology, Xinmofan Road No. 5 Nanjing, Nanjing 210009, People’s Republic of China; b College of Science, Nanjing University of Technolgy, Xinmofan Road No. 5 Nanjing, Nanjing 210009, People’s Republic of China

## Abstract

In the mol­ecule of the title compound, C_11_H_11_FN_2_O_4_, the five-membered ring adopts an envelope conformation. An intra­molecular N—H⋯F hydrogen bond occurs. In the crystal structure, inter­molecular N—H⋯O hydrogen bonds link the mol­ecules.

## Related literature

For general background, see: Kim *et al.* (1994 ▶); Pandey *et al.* (1997 ▶). For bond-length data, see: Allen *et al.* (1987 ▶).
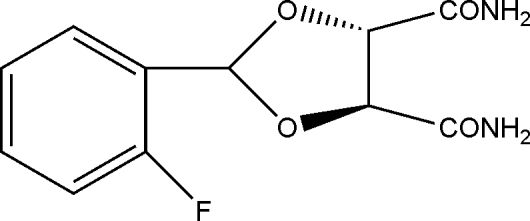


## Experimental

### 

#### Crystal data

C_11_H_11_FN_2_O_4_
*M* *_r_* = 254.22
Orthorhombic, 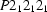

*a* = 4.8760 (5) Å
*b* = 9.1290 (7) Å
*c* = 24.8160 (9) Å
*V* = 1104.63 (15) Å^3^
*Z* = 4
Mo *K*α radiation
μ = 0.13 mm^−1^
*T* = 294 (2) K
0.40 × 0.10 × 0.10 mm

#### Data collection

Enraf–Nonius CAD-4 diffractometer
Absorption correction: ψ scan (North *et al.*, 1968 ▶) *T*_min_ = 0.978, *T*_max_ = 0.987
2157 measured reflections
1301 independent reflections
898 reflections with *I* > 2σ(*I*)
*R*_int_ = 0.072
3 standard reflections frequency: 120 min intensity decay: none

#### Refinement

*R*[*F* ^2^ > 2σ(*F* ^2^)] = 0.060
*wR*(*F* ^2^) = 0.125
*S* = 1.00
1301 reflections
164 parameters
H-atom parameters constrained
Δρ_max_ = 0.27 e Å^−3^
Δρ_min_ = −0.23 e Å^−3^

### 

Data collection: *CAD-4 Software* (Enraf–Nonius, 1985 ▶); cell refinement: *CAD-4 Software*; data reduction: *XCAD4* (Harms & Wocadlo, 1995 ▶); program(s) used to solve structure: *SHELXS97* (Sheldrick, 2008 ▶); program(s) used to refine structure: *SHELXL97* (Sheldrick, 2008 ▶); molecular graphics: *ORTEP-3 for Windows* (Farrugia, 1997 ▶); software used to prepare material for publication: *WinGX* (Farrugia, 1999 ▶).

## Supplementary Material

Crystal structure: contains datablock(s) global, I. DOI: 10.1107/S1600536808039470/hk2584sup1.cif

Structure factors: contains datablock(s) I. DOI: 10.1107/S1600536808039470/hk2584Isup2.hkl

Additional supplementary materials:  crystallographic information; 3D view; checkCIF report

## Figures and Tables

**Table 1:** Hydrogen-bond geometry (Å, °)

*D*—H⋯*A*	*D*—H	H⋯*A*	*D*⋯*A*	*D*—H⋯*A*
N1—H1*A*⋯O4^i^	0.86	2.32	3.089 (4)	149
N1—H1*B*⋯O3^ii^	0.86	2.37	3.164 (4)	153
N2—H2*A*⋯O4^iii^	0.86	2.09	2.944 (5)	172
N2—H2*B*⋯F1	0.86	2.31	3.130 (4)	160

Symmetry codes: (i) 
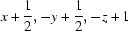
; (ii) 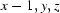; (iii) 
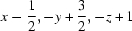
.

**Fig. 1.**
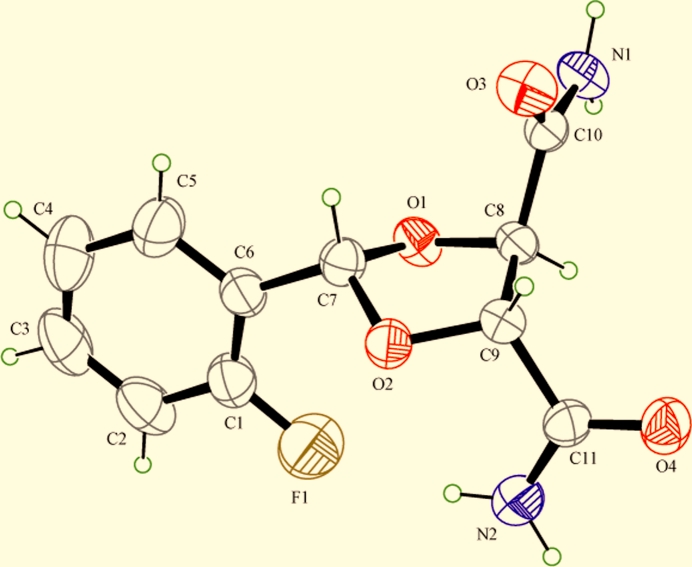
The molecular structure of the title molecule, with the atom-numbering scheme. Displacement ellipsoids are drawn at the 30% probability level.

## supplementary crystallographic information

### Comment

Antitumor platinum drug is one kind of the most effective anticancer agents currently available. (2*S*,3S)-Diethyl 2,3-*O*-alkyltartrate analogues are starting materials for the syntheses of platinum complexes with antitumor activity (Kim *et al.*,  1994), and are also important intermediates in organic syntheses (Pandey *et al.*,  1997). As part of our studies on the syntheses and characterizations of these compounds, we report herein the crystal structure of the title compound.

In the molecule of the title compound (Fig. 1) the bond lengths (Allen *et al.*,  1987) and angles are within normal ranges. The five-membered ring adopts envelope conformation with C7 atom displaced by 0.557 (3) Å from the plane of the other ring atoms. The intramolecular N-H···F hydrogen bond (Table 1) results in the formation of a nine-membered ring: (F1/C1/C6/C7/O2/C9/C11/N2/H2B) having twisted conformation.

In the crystal structure, intermolecular N-H···O hydrogen bonds (Table 1) link the molecules, in which they may be effective in the stabilization of the structure.

### Experimental

For the preparatrion of the title compound, a mixture of 2-fluorbenzaldehyde (302 mg, 2.43 mmol), (2*S*,3S)-diethyltartrate (500 mg, 2.43 mmol), anhydrous copper sulfate (776 mg, 2.86 mmol) and one drop of methanesulfonic acid in anhydrous toluen (8 ml) was stirred at room temperature for 12 h. Anhydrous potassium carbonate (40 mg) was added to the reaction mixture, which was then stirred for a further 20 min. The resulting colorless precipitate was obtained by evaporation and dried in vacuo (yield; 87%). The obtained colorless product (10 mmol) was dissolved in anhydrous ethanol (50 ml), then a current of dry ammonia, dried with calcium chloride passed into the reaction mixture at room temperature for about 6 h. The reaction mixture was evaporated to dryness. Pure compound was obtained by crystallization from dichloromethane. Crystals suitable for X-ray analysis were obatined by slow evaporation of an ethanol solution after one week.

### Refinement

H atoms were positioned geometrically, with N-H = 0.86 Å (for NH_2_) and C-H = 0.93 and 0.98 Å for aromatic and methine H, respectively, and constrained to ride on their parent atoms with U_iso_(H) = 1.2U_eq_(C,N).

### Crystal data

**Table:**

C_11_H_11_FN_2_O_4_	*F*(000) = 528
*M**_r_* = 254.22	*D*_x_ = 1.529 Mg m^−^^3^
Orthorhombic, *P*2_1_2_1_2_1_	Mo *K*α radiation, λ = 0.71073 Å
Hall symbol: P 2ac 2ab	Cell parameters from 25 reflections
*a* = 4.8760 (5) Å	θ = 9–12°
*b* = 9.1290 (7) Å	µ = 0.13 mm^−^^1^
*c* = 24.8160 (9) Å	*T* = 294 K
*V* = 1104.63 (15) Å^3^	Block, colorless
*Z* = 4	0.40 × 0.10 × 0.10 mm

### Data collection

**Table:**

Enraf–Nonius CAD-4 diffractometer	898 reflections with *I* > 2σ(*I*)
Radiation source: fine-focus sealed tube	*R*_int_ = 0.072
graphite	θ_max_ = 26.0°, θ_min_ = 1.6°
ω/2θ scans	*h* = −5→5
Absorption correction: ψ scan (North *et al.*, 1968)	*k* = 0→11
*T*_min_ = 0.978, *T*_max_ = 0.987	*l* = 0→30
2157 measured reflections	3 standard reflections every 120 min
1301 independent reflections	intensity decay: none

### Refinement

**Table:**

Refinement on *F*^2^	Secondary atom site location: difference Fourier map
Least-squares matrix: full	Hydrogen site location: inferred from neighbouring sites
*R*[*F*^2^ > 2σ(*F*^2^)] = 0.060	H-atom parameters constrained
*wR*(*F*^2^) = 0.125	*w* = 1/[σ^2^(*F*_o_^2^) + (0.002*P*)^2^ + 1.775*P*] where *P* = (*F*_o_^2^ + 2*F*_c_^2^)/3
*S* = 1.00	(Δ/σ)_max_ < 0.001
1301 reflections	Δρ_max_ = 0.27 e Å^−^^3^
164 parameters	Δρ_min_ = −0.23 e Å^−^^3^
0 restraints	Extinction correction: *SHELXL97* (Sheldrick, 2008), Fc^*^=kFc[1+0.001xFc^2^λ^3^/sin(2θ)]^-1/4^
Primary atom site location: structure-invariant direct methods	Extinction coefficient: 0.023 (4)

### Special details

**Table:**

Geometry. All e.s.d.'s (except the e.s.d. in the dihedral angle between two l.s. planes) are estimated using the full covariance matrix. The cell e.s.d.'s are taken into account individually in the estimation of e.s.d.'s in distances, angles and torsion angles; correlations between e.s.d.'s in cell parameters are only used when they are defined by crystal symmetry. An approximate (isotropic) treatment of cell e.s.d.'s is used for estimating e.s.d.'s involving l.s. planes.
Refinement. Refinement of *F*^2^ against ALL reflections. The weighted *R*-factor *wR* and goodness of fit *S* are based on *F*^2^, conventional *R*-factors *R* are based on *F*, with *F* set to zero for negative *F*^2^. The threshold expression of *F*^2^ > σ(*F*^2^) is used only for calculating *R*-factors(gt) *etc*. and is not relevant to the choice of reflections for refinement. *R*-factors based on *F*^2^ are statistically about twice as large as those based on *F*, and *R*- factors based on ALL data will be even larger.

### Fractional atomic coordinates and isotropic or equivalent isotropic displacement parameters (Å^2^)

**Table:**

	*x*	*y*	*z*	*U*_iso_*/*U*_eq_	
O1	0.6898 (8)	0.3384 (4)	0.36430 (13)	0.0497 (10)	
O2	0.9373 (9)	0.5438 (3)	0.37339 (12)	0.0489 (10)	
O3	1.1580 (8)	0.2201 (5)	0.44108 (17)	0.0576 (11)	
O4	0.7161 (10)	0.6057 (4)	0.50750 (13)	0.0599 (12)	
N1	0.7627 (11)	0.0954 (4)	0.44684 (15)	0.0513 (12)	
H1A	0.8450	0.0144	0.4540	0.062*	
H1B	0.5866	0.0979	0.4449	0.062*	
N2	0.5605 (12)	0.6946 (5)	0.42850 (18)	0.0681 (16)	
H2A	0.4529	0.7561	0.4441	0.082*	
H2B	0.5674	0.6905	0.3939	0.082*	
F1	0.4814 (11)	0.6150 (5)	0.30682 (15)	0.1054 (16)	
C1	0.6335 (15)	0.5470 (7)	0.2681 (2)	0.0633 (18)	
C2	0.572 (2)	0.5797 (8)	0.2158 (3)	0.083 (2)	
H2	0.4353	0.6472	0.2076	0.099*	
C3	0.715 (2)	0.5109 (8)	0.1758 (3)	0.088 (3)	
H3	0.6700	0.5274	0.1399	0.105*	
C4	0.9265 (19)	0.4170 (9)	0.1887 (2)	0.095 (3)	
H4	1.0286	0.3731	0.1615	0.114*	
C5	0.9875 (19)	0.3878 (8)	0.2426 (2)	0.076 (2)	
H5	1.1281	0.3230	0.2511	0.092*	
C6	0.8410 (14)	0.4542 (6)	0.2832 (2)	0.0539 (16)	
C7	0.9126 (13)	0.4196 (6)	0.34082 (18)	0.0466 (13)	
H7	1.0818	0.3617	0.3423	0.056*	
C8	0.7392 (14)	0.3487 (5)	0.42137 (18)	0.0461 (14)	
H8	0.5644	0.3507	0.4409	0.055*	
C9	0.8871 (13)	0.4968 (5)	0.42764 (19)	0.0470 (14)	
H9	1.0617	0.4826	0.4465	0.056*	
C10	0.9067 (11)	0.2163 (6)	0.4389 (2)	0.0407 (12)	
C11	0.7142 (14)	0.6068 (5)	0.4576 (2)	0.0492 (14)	

### Atomic displacement parameters (Å^2^)

**Table:**

	*U*^11^	*U*^22^	*U*^33^	*U*^12^	*U*^13^	*U*^23^
O1	0.056 (2)	0.0459 (19)	0.0467 (19)	−0.007 (2)	−0.009 (2)	0.0041 (16)
O2	0.059 (2)	0.0420 (18)	0.0451 (19)	−0.007 (2)	0.006 (2)	−0.0011 (15)
O3	0.038 (2)	0.059 (2)	0.075 (3)	−0.006 (2)	−0.003 (2)	0.007 (2)
O4	0.079 (3)	0.054 (2)	0.046 (2)	−0.016 (3)	0.003 (2)	−0.0063 (17)
N1	0.055 (3)	0.044 (2)	0.055 (2)	−0.001 (3)	−0.009 (3)	0.008 (2)
N2	0.093 (4)	0.060 (3)	0.052 (3)	0.020 (4)	0.000 (3)	−0.004 (2)
F1	0.106 (4)	0.128 (4)	0.082 (3)	0.045 (4)	−0.016 (3)	0.000 (3)
C1	0.071 (5)	0.066 (4)	0.054 (3)	0.003 (4)	−0.003 (3)	0.005 (3)
C2	0.106 (6)	0.072 (4)	0.070 (4)	−0.004 (5)	−0.022 (5)	0.017 (4)
C3	0.118 (7)	0.091 (5)	0.053 (4)	−0.021 (6)	−0.021 (5)	0.021 (4)
C4	0.108 (6)	0.134 (7)	0.043 (3)	−0.003 (7)	0.000 (4)	−0.005 (4)
C5	0.088 (5)	0.087 (5)	0.054 (4)	0.010 (5)	0.009 (4)	−0.010 (3)
C6	0.068 (4)	0.048 (3)	0.046 (3)	−0.003 (3)	0.000 (3)	0.005 (2)
C7	0.048 (3)	0.047 (3)	0.045 (3)	0.002 (3)	0.001 (3)	0.000 (2)
C8	0.063 (4)	0.037 (2)	0.038 (3)	0.003 (3)	−0.002 (3)	0.002 (2)
C9	0.056 (4)	0.042 (3)	0.043 (3)	0.001 (3)	0.002 (3)	0.003 (2)
C10	0.045 (3)	0.039 (3)	0.038 (3)	0.001 (3)	0.001 (3)	−0.002 (2)
C11	0.062 (4)	0.039 (3)	0.047 (3)	−0.009 (3)	0.002 (3)	−0.006 (2)

### Geometric parameters (Å, °)

**Table:**

O1—C7	1.438 (6)	C3—C4	1.378 (11)
O1—C8	1.440 (5)	C3—H3	0.9300
O2—C7	1.398 (5)	C4—H4	0.9300
O2—C9	1.434 (5)	C5—C4	1.396 (8)
O3—C10	1.227 (6)	C5—H5	0.9300
O4—C11	1.240 (5)	C6—C1	1.372 (8)
N1—C10	1.323 (6)	C6—C5	1.376 (8)
N1—H1A	0.8600	C7—C6	1.505 (7)
N1—H1B	0.8600	C7—H7	0.9800
N2—H2A	0.8600	C8—H8	0.9800
N2—H2B	0.8600	C9—C8	1.540 (7)
F1—C1	1.363 (7)	C9—H9	0.9800
C2—C1	1.366 (7)	C10—C8	1.522 (7)
C2—H2	0.9300	C11—N2	1.313 (7)
C3—C2	1.367 (10)	C11—C9	1.507 (7)
			
C7—O1—C8	103.8 (4)	C5—C6—C7	118.9 (6)
C7—O2—C9	106.6 (3)	O1—C7—C6	108.5 (5)
C10—N1—H1A	120.0	O1—C7—H7	110.1
C10—N1—H1B	120.0	O2—C7—O1	104.4 (4)
H1A—N1—H1B	120.0	O2—C7—C6	113.5 (4)
C11—N2—H2A	120.0	O2—C7—H7	110.1
C11—N2—H2B	120.0	C6—C7—H7	110.1
H2A—N2—H2B	120.0	O1—C8—C10	108.6 (4)
F1—C1—C2	116.8 (7)	O1—C8—C9	103.6 (4)
F1—C1—C6	119.4 (5)	O1—C8—H8	109.9
C2—C1—C6	123.9 (7)	C9—C8—H8	109.9
C1—C2—C3	118.5 (7)	C10—C8—C9	114.6 (5)
C1—C2—H2	120.8	C10—C8—H8	109.9
C3—C2—H2	120.8	O2—C9—C8	104.3 (4)
C2—C3—C4	119.9 (6)	O2—C9—C11	111.0 (4)
C2—C3—H3	120.0	O2—C9—H9	109.8
C4—C3—H3	120.0	C8—C9—H9	109.8
C3—C4—C5	120.1 (7)	C11—C9—C8	111.9 (5)
C3—C4—H4	120.0	C11—C9—H9	109.8
C5—C4—H4	120.0	O3—C10—N1	123.2 (6)
C6—C5—C4	120.5 (7)	O3—C10—C8	121.8 (5)
C6—C5—H5	119.8	O4—C11—N2	123.9 (6)
C4—C5—H5	119.8	O4—C11—C9	118.9 (5)
C1—C6—C5	117.1 (6)	N1—C10—C8	114.8 (4)
C1—C6—C7	124.1 (5)	N2—C11—C9	117.1 (5)

### Hydrogen-bond geometry (Å, °)

**Table:**

*D*—H···*A*	*D*—H	H···*A*	*D*···*A*	*D*—H···*A*
N1—H1A···O4^i^	0.86	2.32	3.089 (4)	149
N1—H1B···O3^ii^	0.86	2.37	3.164 (4)	153
N2—H2A···O4^iii^	0.86	2.09	2.944 (5)	172
N2—H2B···F1	0.86	2.31	3.130 (4)	160

Symmetry codes: (i) *x*+1/2, −*y*+1/2, −*z*+1; (ii) *x*−1, *y*, *z*; (iii) *x*−1/2, −*y*+3/2, −*z*+1.
